# An outbreak of chickenpox in an asylum seeker centre in Italy: outbreak investigation and validity of reported chickenpox history, December 2015–May 2016

**DOI:** 10.2807/1560-7917.ES.2017.22.46.17-00020

**Published:** 2017-11-16

**Authors:** Francesco Vairo, Virginia Di Bari, Vincenzo Panella, Giuseppe Quintavalle, Saul Torchia, Maria Cristina Serra, Maria Teresa Sinopoli, Maurizio Lopalco, Giancarlo Ceccarelli, Federica Ferraro, Sabrina Valle, Licia Bordi, Maria Rosaria Capobianchi, Vincenzo Puro, Paola Scognamiglio, Giuseppe Ippolito, the Outbreak Investigation Group

**Affiliations:** 1 National Institute for Infectious Diseases INMI ‘L. Spallanzani’ IRCCS, Rome, Italy; 2 Regional Service for Infectious Diseases Surveillance and Response (SERESMI), Latium Region, Rome, Italy; 3 These authors contributed equally to the manuscript; 4 Directorate of Health and Social Welfare, Latium Region, Rome, Italy; 5 Local Public Health Unit ASL Roma 4, Latium Region, Rome, Italy; 6 Sanitary Bureau of Asylum Seekers Center of Castelnuovo di Porto, Rome, Italy; 7 Auxilium Società Cooperativa Sociale, Senise (Potenza), Italy; 8 The members of the Outbreak Investigation Group are listed at the end of the article

**Keywords:** varicella, chickenpox, outbreaks, surveillance, Italy, migrant

## Abstract

An outbreak of chickenpox occurred between December 2015 and May 2016 among asylum seekers in a reception centre in Latium, Italy. We describe the epidemiological and laboratory investigations, control measures and validity of reported history of chickenpox infection. Serological screening of all residents and incoming asylum seekers was performed, followed by vaccine offer to all susceptible individuals without contraindication. Forty-six cases were found and 41 were associated with the outbreak. No complications, hospitalisations or deaths occurred. Serological testing was performed in 1,278 individuals and 169 were found to be susceptible, with a seroprevalence of 86.8%. A questionnaire was administered to 336 individuals consecutively attending the CARA health post to collect their serological result. The sensitivity, specificity and the positive and negative predictive value (PPV and NPV) of the reported history of chickenpox were 45.0%, 76.1%, 88.3% and 25.6%, respectively. We observed an increasing trend for the PPV and decreasing trend for the NPV with increasing age. Our report confirms that, in the asylum seeker population, chickenpox history is not the optimal method to identify susceptible individuals. Our experience supports the need for additional prevention and control measures and highlights the importance of national and local surveillance systems for reception centres.

## Background

The International Organisation for Migration estimated that 345,440 migrants and refugees entered Europe by sea in 2016 up to 22 November (171,264 in Greece and 168,542 in Italy) [1]. In March 2016, the Italian Ministry of Interior estimated that ca 111,000 migrants were living in Italy and most of them were asylum seekers living in collective housing facilities [2]. The term ‘migrant’ as used in this paper covers also refugees and asylum seekers. The migrant population is usually made up of young and healthy people, who are at risk for infectious diseases as a consequence of the difference in infectious disease prevalence between their countries of origin and the hosting countries as well as the conditions they experience during migration [3]. An additional risk is posed by the specific challenges faced by collective housing facilities in preventing and controlling communicable disease transmission in ‘semi-open’ communities [4] with an often higher than affordable number of people entering the facilities. Among the infectious diseases potentially affecting the migrant population, chickenpox (mainly transmitted through the airborne route) is characterised by a high potential of spread in closed and semi-open communities, and by a slightly lower seroprevalence in tropical compared with temperate areas: In temperate regions, ca 95% of people 12 years and older are immune [5,6] while in tropical areas, seroprevalence in adults varies from 93.5% to 70%, with a proportion of susceptible individuals ranging from 6.5% to 30% [7,8]. Several outbreaks of chickenpox among asylum seeker populations have been reported in the literature [7,9-11]. Vaccination of susceptible individuals was a key intervention for outbreak control [6,7].

In 2011, the Italian Ministry of Health (MoH) has implemented a syndromic surveillance system in collective housing facilities in order to rapidly detect potential public health emergencies [12]. The syndromic surveillance system works in parallel with the surveillance system for notifiable infectious diseases which was strengthened in 2015 in the Latium region to respond to the expected high influx of people visiting Rome for the one-year Jubilee 2015–16, one of the most important Catholic events that sees pilgrims gather in Rome to pray) [13].

Within the framework of the enhanced surveillance, the Lazio Regional Service for the epidemiology and control for infectious diseases (SERESMI) was alerted in January 2016 by the syndromic surveillance system of a cluster of ‘fever with rash syndrome’ among the population living in an asylum seekers centre (CARA), followed by notifications of five cases of chickenpox. The CARA health staff and the local public health authority promptly implemented all routine control measures (isolation of cases, contact tracing and vaccination of close contacts with negative chickenpox history). Despite these control interventions, 25 more cases were notified during the following 3 months. In April 2016, new control measures were recommended and implemented: serological screening of all residents and incoming asylum seekers followed by vaccine offer to all susceptible individuals without contraindications (immunodepression, pregnancy, etc). During the implementation phase of the additional control measures, 16 new cases were notified. The last case was notified on 17 May 2016. 

This report describes the epidemiological and laboratory investigations and the control measures implemented during the outbreak. In order to evaluate the performance of reported history to assess immune status, a questionnaire was administered to a subgroup of serologically screened individuals.

## Methods

### Setting

CARA centres are facilities hosting newly arriving migrants who seek international protection; they were established following the reform of the asylum law, enacted to implement two European Union (EU) directives [14,15]. They are under the authority of the Ministry of Interior through the Prefectures which entrust the management to private or non-governmental bodies. The CARA involved in the outbreak is located in Rome and is the largest in the Latium Region. Before the outbreak, the population residing in the centre was made up of young (median age: 25 years; range: 18-60 years) and almost exclusively male (93%) individuals coming mainly from Mali (20%), Nigeria (15%), Gambia (13%), Senegal (11%) and Pakistan (11%).

During the outbreak, the previous CARA setting changed following accelerated implementation of the EU relocation and resettlement scheme for refugees and asylum seekers (providing a mandatory and automatically triggered relocation system to distribute those in clear need of international protection within the EU when a mass migration occurs) [16]. The median length of stay in the centre became shorter and the population came mainly from Eritrea (59%).

### Case definition

Confirmed cases were defined as cases that met the clinical criteria and were laboratory-confirmed by detection of specific IgM antibodies against chickenpox virus, or as cases that met the clinical criteria and were epidemiologically linked to another probable or confirmed case, even in the absence of laboratory confirmation [17].

At screening, people with reactive serological tests were defined as immune whereas people with a non-reactive or indeterminate test were categorised as susceptible.

### Outbreak investigation and control

From January to March 2016, all identified cases were immediately isolated and offered oral acyclovir medication. Contact tracing and vaccination of close contacts with negative chickenpox history and active case finding were implemented and the epidemiological link was assessed for all cases occurring during the outbreak period.

Starting in April 2016, all residents and all new arrivals at the CARA up to 42 days after the last case were tested for VZV antibodies using chemiluminescence immunoassay (CLIA) technology on the LIAISON XL analyser (DiaSorin S.p.A., Vercelli, Italy) for the quantitative detection of specific VZV IgG and IgM antibodies.

Before vaccination, HIV testing (ARCHITECT HIV Ag/Ab Combo assay; Abbott Diagnostics, Santa Clara, United States (US)) was offered to all chickenpox-susceptible individuals and pregnancy tests were performed in all chickenpox-susceptible women. Susceptible individuals without contraindications such as pregnancy or immunosuppression were offered chickenpox immunisation (OKA vaccine strain) with the routine two-dose schedule. The contacts of the initial cases were offered and received vaccination and were not included in the serological screening. The outbreak was considered over after two full incubation periods (42 days) had passed since the last case was identified.

### Validity of reported chickenpox history

All people consecutively attending the CARA health post to collect their serological result before the vaccination session were asked to participate in the validity analysis of chickenpox infection history. A structured questionnaire was administered, in a face-to-face structured interview, to those who consented to participate. The interviews were conducted after the participant signed the informed consent form and before they collected their serological results. All data contained in the manuscript were obtained during the epidemiological investigation as an institutional duty of the Latium Regional Health Authority (RHA), in order to identify/contain an ongoing epidemic cluster, to provide recommendations, to prevent new outbreaks and to avert complications in infected subjects. The approval of the National Institute for Infectious Diseases Spallanzani's Institutional Review Board was not required since we operated under emergency circumstance.

Interviews were conducted in English or French, or in the participants’ native languages with the aid of the cultural mediators working in the CARA. The questionnaires gathered information on demographic data, education, countries crossed during the migration journey to reach Italy with length of stay and housing conditions in each crossed country, and history of chickenpox infection. This latter information was gathered through four different questions with increasing specificity and with the aid of images of chickenpox skin eruptions. Answers were categorised as ‘yes’, ‘no’ or ‘unknown’. Individuals with a positive answer to any question regarding chickenpox history were classified as having a positive chickenpox history. Assuming that clinicians would opt to immunise patients with an ‘unknown’ answer, individuals with negative and/or unknown answers were classified as having negative chickenpox history.

### Data collection and statistical analyses

Data on the outbreak cases were collected from the notification forms and patients’ medical records. Data on the screened individuals were obtained from patients’ medical records. The median length of stay at CARA was calculated by subtracting the date of arrival from the date of the serological testing. The attack rate was calculated among the average population resident at the CARA during the outbreak period and assuming that all residents were exposed. Prevalence of chickenpox susceptibility was calculated among all residents and all new arrivals at the CARA from the date of onset of the first case up to 42 days after the date of onset of the last case. Descriptive statistics were calculated by means of proportion, mean and median. Associations between serological status and demographic variables were assessed by chi-squared test for proportions and by t-test for means.

The validity of the questionnaire for evaluation of chickenpox history was evaluated through calculation of sensitivity, specificity, positive predictive value (PPV), negative predictive value (NPV) and relative 95% confidence intervals (CI) using binomial distribution. The likelihood-ratio test was used to further estimate PPV and NPV, assuming that the exact prevalence was the prevalence among all screened individuals. Among the interviewed patients, age groups were built based on the quartiles of the age distribution.

All statistical analyses were performed using STATA software version 14.

## Results

### The outbreak investigation

From December 2015 to June 2016, 46 cases of chickenpox occurred among the asylum seeker population living at the CARA (Figure).

**Figure:**
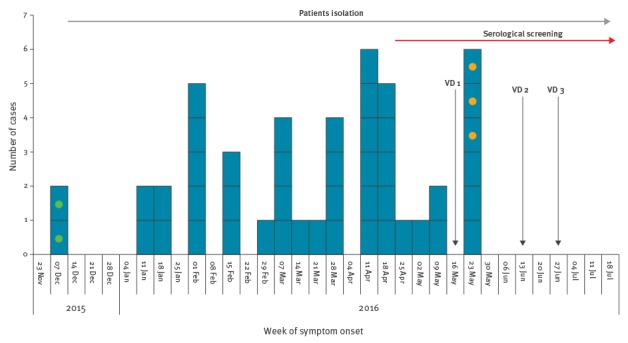
Epidemiological curve and outbreak control measures, Italy, December 2015−May 2016 (n = 46)

VD: vaccination day.

Green dots: cases involved in two probably separate cases in early December 2015; yellow dots: probably imported cases.

All cases were identified through clinical examination by medical practitioners working in the centre. Of the 46 cases, two cases in early December 2015 (Figure, green dots) were retrospectively identified as a possible separate cluster. Three cases in 2016 (Figure, yellow dots). were considered imported cases because the dates of check-in and symptom onset were not compatible with the acquisition of the disease at the CARA (incubation period: 10–21 days) and because all three reported a history of contact with people with possible chickenpox skin manifestations before their arrival at the Centre. Forty-one cases were associated with the main outbreak. Among the 41 outbreak cases, median age was 26 years (interquartile range: 21–29) and 40 cases were male. The majority of patients were born in Eritrea (11/41), Nigeria (10/41), followed by Mali (7/41), Ghana (5/41) and Ivory Coast (2/41), Kuwait (2/41), Pakistan (2/41), Equatorial Guinea (1/41) and Togo (1/41). The time between their arrival in the CARA and the onset of symptoms ranged from 10 days to 3.9 years. The overall attack rate among the calculated among the average population of 837 residents at the CARA during the outbreak period was 4.9%. No complications, hospitalisations or deaths occurred. No cases occurred among the 112 staff in the CARA. 

### Chickenpox seroprevalence

Serological testing was performed in 1,278 individuals who resided in the CARA or arrived there during the outbreak period (Table 1). Among them, 169 individuals were susceptible to chickenpox, which corresponds to a prevalence of 13.2% (169/1,278). The majority of the 1,278 tested individuals (1,048/1,278; 82%) were male and the mean age was 25 years (standard deviation (SD): ± 7). Most of the patients were from East Africa (60%) and West Africa (29%); Eritrea was the most represented country (59%). In the univariate analysis, age was significantly associated with the serological immune status (p = 0.02) with an increase of 4% in the odds ratio (OR) of being immune for each year of increase in age (OR = 1.04; 95% confidence interval (CI): 1.01–1.07).

**Table 1:** Socio-demographic characteristics of serologically screened individuals, by chickenpox immune status, Italy, December 2015−May 2016 (n = 1,278)

	Immune (n = 1,109)		Susceptible (n = 169)		Total population (n = 1,278)		p value
	n	%	n	%	n	%	
Sex							
Male	909	82.0	139	82.2	1,048	82.0	0.96 ^a^
Female	198	17.8	30	17.8	228	17.8	
Missing	2	0.2	0	0	2	0.2	
Age (years) as mean (± SD)	25 (± 7)		24 (± 5)		25 (± 7)		0.02 ^b^
Length of stay in the centre in days, median (IQR)	5 (1 – 222)		4 (1 – 222)		5 (1 – 222)		0.81
Country of birth							
Eritrea	646	58.3	109	64.5	755	59.1	0.65 ^a^
Nigeria	71	6.4	14	8.3	85	6.7	
The Gambia	65	5.9	4	2.4	69	5.4	
Mali	59	5.3	9	5.3	68	5.3	
Pakistan	47	4.2	11	6.5	58	4.5	
Senegal	53	4.8	5	3.0	58	4.5	
Guinea	35	3.2	2	1.2	37	2.9	
Bangladesh	30	2.7	4	2.4	34	2.7	
Ghana	26	2.3	4	2.4	30	2.3	
Syria	23	2.1	1	0.6	24	1.9	
Côte d’Ivoire	12	1.1	2	1.2	14	1.1	
Sudan	9	0.8	2	1.2	11	0.9	
Ethiopia	10	0.9	0	0	10	0.8	
Togo	6	0.5	0	0	6	0.5	
Guinea-Bissau	4	0.4	0	0	4	0.3	
Burkina Faso	2	0.2	0	0	2	0.2	
Palestine ^c^	2	0.2	0	0	2	0.2	
Benin	1	0.1	0	0	1	0.1	
Cameroon	1	0.1	0	0	1	0.1	
Congo	1	0.1	0	0	1	0.1	
India	0	0	1	0.6	1	0.1	
Iraq	1	0.1	0	0	1	0.1	
Liberia	1	0.1	0	0	1	0.1	
Central African Republic	0	0	1	0.6	1	0.1	
Sierra Leone	1	0.1	0	0	1	0.1	
Sri Lanka	1	0.1	0	0	1	0.1	
Missing	2	0.2	0	0	2	0.2	
Geographic region							
East Africa	656	59.2	109	64.5	756	59.9	0.06 ^a^
West Africa	336	30.3	40	23.7	376	29.4	
South Asia	78	7.0	16	9.5	94	7.4	
West Asia	26	2.3	3	1.8	27	2.1	
Central Africa	11	1.0	1	0.6	14	1.1	
Missing	2	0.2	0	0	2	0.2	

IQR: interquartile range; SD: standard deviation.

^a^ Chi-squared test.

^b^ t-test.

^c^ This designation shall not be construed as recognition of a State of Palestine and is without prejudice to the individual positions of the Member States on this issue.

### Outbreak control

Among the 169 susceptible individuals, five (2.9%) had vaccination contraindications, 41 (24%) declined vaccination and eight (5%) were relocated before the first vaccination session. Of the remaining 115 individuals, 54 (47%) received only one dose and 61 (53%) completed the two-dose vaccination schedule. Of 54 individuals who did not complete the two-dose vaccination schedule, information regarding the reason for incomplete schedule was available for 52 individuals. Of these, 28 were relocated before the second dose, and 24 declined the second dose.

Time of vaccination is shown in the Figure. Rapid displacement within national and international territories and refusal of vaccination were the main hindrances to vaccination access. One patient who received the first dose of chickenpox vaccine developed a varicella-like rash 5 days after the vaccination.

As the last case occurred on 17 May, the outbreak was considered ended on 28 June 2016.

### Validity analysis of chickenpox infection history

Of the 1,278 serologically screened individuals, 336 (26.3%) were interviewed and included in the validity analysis. Of the 336 interviewed individuals, 173 (51%) reported a negative chickenpox history and 47 (27%) of these were serologically susceptible. A total of 137 individuals (41%) reported having had chickenpox and 121 of them (88%) were serologically immune. Finally, 26 of the 336 (7.7%) reported an unknown history of chickenpox infection; four of these had negative serology. For the purpose of this analysis, we considered reported unknown histories as negative histories. Subjects reporting positive history of chickenpox were older (p < 0.001) and more likely to come from urban areas (p < 0.001); the same distribution was observed in serologically immune subjects (Table 2).

**Table 2:** Characteristics of individuals included in the validity analysis, by chickenpox serological immune status and chickenpox history, Italy, December 2015−May 2016 (n = 336)

	Total		Serological test					p^a^	Questionnaire								p^a^
			Immune			Susceptible			Immune				Susceptible				
			n		%	n	%		n			%	n	%			
Sex																	
Male	268	79.8	221	82.5		47	17.5	0.02	106		39.6		162		60.4		0.36
Female	67	19.9	47	70.2		20	29.9		30		44.8		37		55.2		
Missing	1	0.3	1	100		0	0		1		100		0		0		
Age (years) as mean (± SD)	24.5	6.1	25.5 (6.3)			23.3(4.7)		0.001	26.6(6.8)				23.9(5.2)				< 0.001^b^
Geographic region																	
East Africa	223	66.4	177	79.4		46	20.6	0.242	100		44.8		123		55.2		0.123
West Africa	79	23.5	67	84.8		12	15.2		23		29.1		56		70.9		
South Asia	18	5.4	11	61.1		7	38.9		7		38.9		11		61.1		
West Asia	5	1.5	5	100		0	0		3		60.0		2		40.0		
Central Africa	10	3.0	8	80.0		2	20.0		3		30.0		7		70.0		
Missing	1	0.3	1	100		0	0		1		100		0		0		
Area																	
Urban	155	46.1	132	85.2		23	14.8	0.02	82		52.9		73		47.1		<0.001
Rural	165	49.1	122	73.9		43	26.1		49		29.7		116		70.3		
Missing	16	4.8	15	93.7		1	6.3		6		37.5		10		62.5		
Education																	
No education	28	8.3	27	96.4		1	3.6	0.07	9	32.1			19			67.9	0.607
Any	300	89.3	236	78.7		64	21.3		125	41.7			175			58.3	
Missing	8	2.4	6	75.0		2	25.0		3	37.5			5			62.5	

SD: standard deviation.

^a^ Chi-squared test.

^b^ t-test.

The sensitivity and specificity of reported history of chickenpox were 45% (95% CI: 39–51) and 76.1% (95% CI: 65.9–86.3), respectively; PPV and NPV were 88.3% (95% CI: 82.9–93.7) and 25.6% (95% CI: 19.6–31.7), respectively (Table 3).

**Table 3:** Sensitivity, specificity, postitive and negative predictive value of history of chickenpox infection, by prevalence and age groups, Italy, December 2015−May 2016 (n = 336)

	Seroprevalence %	Sensitivity **% (95% CI)**	Specificity **% (95% CI)**	PPV **% (95% CI)**	NPV **% (95% CI)**
Total population	80.1	45.0 (39–51)	76.1 (65.9–86.3)	88.3 (82.9–93.7)	25.6 (19.6–31.7)
Age groups					
≤ 20 years	68.8	34.5 (22.0–47.1)	84.0 (69.6–98.4)	82.6 (67.1–98.1)	36.8 (24.3–49.4)
21–23 years	79.8	32.8 (21.6–44.1)	70.6 (48.9–92.2)	81.5 (66.8–96.1)	21.1 (10.5–31.6)
24–27 years	85.1	50.0 (38.6–61.4)	84.6 (65.0–100)	94.9 (87.9–100)	22.9 (11.0–34.8)
> 28 years	85.9	58.9 (47.6–70.2)	58.3 (30.4–86.2)	89.6 (80.9–98.2)	18.9 (6.3–31.5)
Assuming prevalence = 86.8%	86.8	NA	NA	92.5 (88.8–95.1) ^a^	17.4 (15–20) ^a^

NA: not applicable; NPV: negative predictive value; PPV: positive predictive value.

^a^ Calculated using likelihood ratio test.

Age-stratum sensitivity, specificity, PPV and NPV are reported in Table 3. An increasing sensitivity was observed with increasing age groups. Conversely, specificity showed a decreasing trend except for the oldest age group. PPV was generally stable and NPV showed a decreasing trend with increasing age.

Moreover, sensitivity was higher in individuals coming from urban areas compared with rural (54.5% vs 35.2%), while specificity and NPV were lower (56.5% vs 86% and 17.8% vs 31.9%, respectively).

## Discussion

### Outbreak investigation and control

Asylum seekers centres and other residential institutions are crowded and semi-open communities in which transmission of chickenpox is likely to occur. Residents and staff are at high risk of severe disease and complications because of their adult age [3]. Once a case occurs, control measures such as strict isolation of the patient and contact tracing are often difficult to implement and the case can become a potential source of an outbreak. The epidemic event we reported here clearly suffered from these difficulties: despite the initial prompt isolation and treatment of cases and the contact tracing measures, 41 outbreak cases of chickenpox were documented. It is likely that the use of common facilities (e.g. dining room, laundry areas and recreational areas) during the infectious period before the rash onset was the main factor influencing the spread of chickenpox in the centre. Although specific information on possible barriers was not collected, we can hypothesise that language, cultural and relational barriers might have hindered the access to the available healthcare service at CARA. Poor communication and inability to overcome language and cultural barriers seemed to be the most important cause affecting the access to healthcare also in a recent qualitative research study in Sweden [18]. Similar findings were reported by Graetz et al. [19] in a systematic literature review of the use of health services by migrants in Europe.

The outbreak showed an attack rate of 4.9%, a slightly higher rate than that reported in other outbreaks of chickenpox in housing facilities: those for asylum seekers in Switzerland (2.8%) [19], adults in long-term healthcare facility (2.3%) [20], African migrants living in close communities (4.4%) [21] and prisoners in Italy (3.7%) [22]. The attack rate was much lower than that reported in household contacts (> 80%) [23]. This result should however be taken with caution because the exact number of exposed subjects is uncertain owing to the continuous movement of individuals in and out of the centre.

There were no complications related to the disease, and the hospitalisation rate was 0%. The absence of complications could be explained by the prompt offer of acyclovir treatment to all cases and by the generally healthy state of the resident population (healthy migrant effect). 

The first dose of vaccine was administered to 68% of the susceptible population, but only 36% of them completed the two-dose schedule. Nevertheless, virus circulation stopped within a few days of vaccine administration, probably because of its high vaccine effectiveness which is estimated to be 81% (95% CI: 78–84%) after the first dose, increasing to 98% (95% CI: 97–99%) after the second dose [24]. One case had a possible adverse event (rash) to vaccination, probably related to with the vaccine strain [25,26].

A considerable proportion (24%) of susceptible individuals refused to be vaccinated, but data on their reasons for refusal were not systematically collected. The refusal rate could be related to the general low confidence of the immigrant population in the health service as reported in other studies considering the access to health services [18,19].

No data on chickenpox seroprevalence in asylum seeker populations living in Italy are available to date. During the management of this outbreak, we found a chickenpox seroprevalence of 87%. This latter value is consistent with recent published values of 12.5% among asylum seekers in Lower Saxony, Germany [27] but much higher than recent estimates from six northern German reception centres in 2015 (3.3%) [28] and in Canada in 2014 (7.9%) [7]. These differences could be explained by variations in the residents’ age, sex or countries of origin and by differences in migration routes: Italy is often the first asylum country and chickenpox seroprevalence would not be influenced by having lived in other high prevalence countries. The association between chickenpox seroprevalence and increasing age reflects common population age-related patterns of diseases inducing lifelong immunity.

### Validity of reported chickenpox history

When compared with the serological test, history of chickenpox infection showed a low sensitivity (45%; 95% CI: 39–51) and NPV (26%; 95% CI: 20–32) in predicting past infection. The sensitivity we found was slightly lower than that reported in refugees in the US (58%) [29] and much lower than that reported among US military personnel (90%) [30]. The NPV was otherwise in line with findings from refugees and young adults in the US (29% and 23–44%, respectively) [30,31].

Also specificity (76%; 95% CI: 66–86%) and PPV (88%; 95% CI: 83–94%) were in line with that reported in refugees in the US (PPV: 88%), but lower than in young adults in the US (PPV, 98%) [29,30]. Published data on the validity of reported chickenpox history are scarce and mainly stem from studies conducted among populations from Europe and North America. The observed PPV differs between studies probably because of the different seroprevalences of the study populations. The high seroprevalence found in US military personnel (90–96%) could explain the higher PPV compared with our population [30]. The NPV did not show was low also at higher seroprevalence (in young US military personnel [30], the NPV was 23%, with a seroprevalence of 96%). The increasing trend in PPV and decreasing trend in NPV with increasing age in our study could be related to the high chickenpox seroprevalence of 85% in adults older than 24 years. Overall, the validity of the reported history of chickenpox infection we estimated is consistent with published literature that evaluates it as high in children but variable in adolescents and adults and low in refugees [29-32].

Currently, serological screening rather than preventative vaccination is advised for adolescents and adults with a negative or uncertain history of chickenpox, while recommendations differ for those with a positive history of chickenpox, depending on their particular risk of infection [32]. Indeed, if we had used chickenpox history alone to identify susceptible individual in our sample of 336 asylum seekers, we would have vaccinated 148 immune individuals, exposing them to the unjustified risk of side effects, and 16 susceptible individuals who reported a positive history would have been missed. Assuming that the exact seroprevalence was 86.8% (seroprevalence among all serologically screened) the PPV would increase to 92.5% and the NPV would decrease to 17.4%. If we had used chickenpox history alone to identify susceptible individuals among all screened individuals, we would have vaccinated 757 individuals, 625 of whom were immune, and we would have missed 39 susceptible individuals.

The homogeneity of people living at the CARA, with a majority coming from Sub-Saharan Africa, is a constraint on the generalisation of our findings to older and multi-ethnic migrant populations.

Recall bias in relation to age could have impacted the results, especially for those who contracted chickenpox during infancy and early childhood. We attempted to control for recall bias by adding pictures of typical chickenpox rashes to the questionnaires. Considering individuals with an ‘unknown’ answer as negative history of diseases, could have affected the PPV and the NPV.

## Conclusions

In conclusion, the presented outbreak confirms the risk of chickenpox among migrant populations and supports the need for additional measures to prevent and control an outbreak, in order to avoid complications, limit management costs of cases and prevent the further spread of the virus. NPV and PPV indicate that serological testing is crucial for those reporting a negative history of chickenpox and should be considered in those reporting a positive history. Nevertheless, considering that the asylum seeker population is at increased risk of varicella, a universal screening of all individuals, regardless of history status, should be the preferred approach considering associated costs case by case. This report also highlights the important role of national and local surveillance systems for reception centres for migrants in early detection and response to chickenpox and other communicable disease outbreaks and the value of a coordinated response integrating collective housing facilities, public health authorities, reference laboratories and high level specialist hospitals.

## Notes

**Members of the Outbreak Investigation Group:** Keh-Buma Dinga Charles, Valeria Tognoni, Mariarosaria Cuomo, Giada Bellanca, Serena Vita, Ida Walters, Veronica Di Renzo, Irene Conti Papuzza, Maria Leonardi, Elsa Dimitrova, Mihaela Bujor, Monica Sane-Schepisi, Simone Lanini, Roberto Leone, Alessandro Agresta, Carla Nisii, Eleonora Lalle, Luisa Felici, Isabella Abate.
